# Classic Bartter syndrome complicated with profound growth hormone deficiency

**DOI:** 10.1186/1687-9856-2013-S1-P40

**Published:** 2013-10-03

**Authors:** Masanori Adachi, Toshihiro Tajima, Koji Muroya, Yumi Asakura

**Affiliations:** 1 Kanagawa Children's Medical Center, Japan; 2 Hokkaido University School of Medicine, Japan

## Case history

A Japanese boy was born at 41 weeks of gestational age by spontaneous cephalic delivery, with birth weight 3,680g. Family history was remarkable for his elderly sister suffering from classic Bartter syndrome. He also was diagnosed as classic Bartter syndrome, based on the findings such as failure to thrive, metabolic alkalosis with hypokalemia, and the activated renin-aldosterone system. The diagnosis was later confirmed by CLCNKB gene analysis which revealed compound heterozygous mutations: deletion of the exon 1 - 3 (derived from his mother) and ΔL130 (derived from the father). Treatment with spironolactone, indomethacin, sodium chloride, and potassium supplementation was commenced at 8 months of age. However, his serum potassium level failed to normalize, and tended to be around 2 - 3mEq/L. He had mild intellectual impairment, and needed specialized education. When he was 11 years old, through the investigation for macrohematuria, renal stones with nephrocalcinosis were detected. This complication resolved following the amelioration of hypokalemia, which was achieved by vigorous potassium fortification. Because his growth rate was consistently subnormal and he was very short (-4.7SD), scrutiny for growth hormone (GH) secretion was conducted at his 14 years of age.

## Results

Profound GH deficiency was evident. Serum IGF-1 level was 80ng/mL (norm, 178-686), with IGFBP-3 1.92μg/mL (2.69-4.16). Pharmacologically stimulated GH levels were 0.15 and 0.39 ng/mL, after insulin and arginine administration, respectively. His bone age was 11.4 years old (TW2-RUS for Japanese). MR imaging detected no abnormality within hypothalamic-pituitary region.

## Clinical course

GH treatment with 0.15-0.19mg/kg/week resulted in growth rate as much as 11.7cm during the first 12-months, followed by 10.8cm during the subsequent 12 months. Although his pubertal stage progressed from Tanner stage 1 to stage 2 during 2 years, Δ bone age / Δ chronological age was 1.02. No significant change in serum potassium level was observed.

## Discussion

In literature, GH deficiency in Bartter syndrome and Gitelman syndrome has been anecdotally reported, with diverse severity among patients. At present, its precise pathogenesis, as well as its prevalence, is unknown. Classic Bartter syndrome complicated with profound GH deficiency, as seen in this case, is rare. The sister of the case, who also suffered from classic Bartter syndrome, is 145 cm tall (-2.0SD) now and seemed not to be GH deficient. Her potassium level had been managed rather better than this case. It may be tempting to speculate that long-term hypokalemia may be harmful for somatotrophs to produce GH.

